# An Explainable Hybrid Machine Learning Approach Based on MPSO-XGBoost for Pest Density Prediction in Almond Orchards

**DOI:** 10.3390/insects17100996

**Published:** 2026-09-25

**Authors:** Cebrail Barut, Doygun Demirol, Harun Bingöl, Hande Yüksel, Bilal Alatas, İnanç Özgen

**Affiliations:** 1 Department of Continuing Education Center, Firat University, Elazig 23119, Türkiye; cbarut@firat.edu.tr; 2 Department of Computer Technologies, Bingöl University, Bingöl 12000, Türkiye; ddemirol@bingol.edu.tr; 3 Department of Software Engineering, Faculty of Engineering and Naturel Sciences, Malatya Turgut Özal University, Malatya 44200, Türkiye; harun.bingol@ozal.edu.tr; 4 Department of Software Engineering, Faculty of Engineering, Firat University, Elazig 23119, Türkiye; hande.yuksel@firat.edu.tr (H.Y.); balatas@firat.edu.tr (B.A.); 5 Department of Bioengineering, Firat University, Elazig 23119, Türkiye

**Keywords:** almond production, pest density prediction, XGBoost, Explainable Artificial Intelligence (XAI), rule discovery

## Abstract

Accurate prediction of pest density and population levels in almond production is of great importance for crop yield, the prevention of quality losses, and the effectiveness of integrated pest management (IPM) strategies. Complex feature interactions in agricultural field data can affect both the predictive performance and interpretability of machine learning models. In this study, an explainable hybrid machine learning framework combining Mutated Particle Swarm Optimization (MPSO), XGBoost classification, and rule extraction was developed to classify pest insect density levels. The proposed approach was evaluated using two complementary validation settings. First, it was compared with 16 classification algorithms, including ensemble, deep learning, and rule-based methods, under stratified 5-fold cross-validation, achieving a mean accuracy of 79.63% and a mean Weighted F1-Score of 79.79%. In addition, nested stratified 5-fold cross-validation was employed to separate MPSO-based hyperparameter optimization from outer-fold performance evaluation. Under this more rigorous evaluation, the proposed approach achieved a mean accuracy of 79.27 ± 1.66% and a mean Weighted F1-Score of 79.29 ± 1.38%. Furthermore, interpretable IF–THEN decision rules were derived from the trained XGBoost decision structures to provide a transparent representation of the feature–threshold combinations associated with the classification decisions.

## Simple Summary

Predicting pest density in almond orchards is essential for preventing crop damage and reducing unnecessary pesticide use. This study introduces an explainable hybrid machine learning approach that integrates MPSO-based hyperparameter optimization, XGBoost classification, and rule extraction to classify pest density levels (low, medium, or high) by evaluating weather data, seasonal timing, and tree growth characteristics. The proposed approach reached approximately 80% accuracy in comparisons with 16 machine learning algorithms tested under the same conditions. Crucially, the model translates complex predictions into clear “IF–THEN” rules based on thresholds such as tree age and temperature, allowing agricultural experts to examine the conditions underlying model decisions.

## 1. Introduction

The almond is a type of hard-shelled fruit with high economic value that is grown in many regions of the world, particularly in the Mediterranean climate zone [1,2]. To sustain high yields and product quality in almond production, growing conditions must be properly managed, and diseases and pests must be effectively controlled. In this context, insect pests can cause damage to leaves, shoots, and fruits during different stages of the tree’s development, leading to a reduction in yield and quality [1,3]. The extent of this damage varies depending on the density of insect pests in orchards [1]. Numerous insect species have been recorded as pests in almond orchards worldwide, and the level of damage they cause to leaves, shoots, and fruit varies depending on the species, phenological stage, and population density [1,4]. It has been reported that pest species capable of developing significant populations in almond orchards are also present in Türkiye’s almond production areas, and the timing of control measures should be based on population monitoring [5]. Pest density varies depending on environmental conditions, seasonal changes, and the structural characteristics of the trees [6,7]. Failure to identify these changes in pest density promptly can lead to delays in control measures and, consequently, increased crop losses. Therefore, it is necessary not only to determine the current density of pests but also to predict high-risk periods by evaluating the variables that affect density. Consequently, the accurate and timely prediction of pest populations is of great importance for implementing control measures at the appropriate time, reducing crop losses, and using production resources more efficiently.

The development and distribution of insect pests in orchards are closely related not only to their biological characteristics but also to the environmental conditions in which they live [6,7]. In particular, temperature and relative humidity can influence the insects’ development period, survival, and reproductive activities, leading to seasonal fluctuations in population density [6,7]. In addition, wind speed, elevation, and habitat characteristics can affect the movement and distribution of pests within orchards [8,9,10]. In fact, it has been shown that the increase in temperature directly changes the development rate, generation number and first appearance time of harmful insects in the season, which increases pest pressure in hard-shelled fruit species such as almonds [11]. In a machine learning-based study conducted under different microclimate conditions, it was determined that temperature is the dominant determinant, while humidity and wind have a secondary effect [8]. Changes in pest density are not limited solely to environmental conditions. Structural characteristics such as tree age, height, and canopy width can alter pests’ feeding and sheltering conditions, leading to variations in density levels among trees. This situation leads to a divergence in density values in samples taken from different parts of the tree within the same orchard; it has been reported that sampling carried out in different layers of the crown structure yields significantly different results [12]. Studies conducted in Mediterranean garden systems have shown that changes in tree density, age, and size determine the abundance of canopy arthropods [13]. In addition, it has been observed that the species diversity detected in almond orchards can vary greatly between orchards, which is associated with structural and environmental differences at the orchard level [2]. Since the effects of these variables do not occur at the same level during different stages of the growing season, temporal characteristics must also be considered when assessing pest density. Indeed, previous studies have shown that the combined evaluation of meteorological variables and plant phenology contributes to the prediction of pest populations [14,15]. Therefore, the reliable prediction of pest density depends on evaluating environmental, temporal, and tree-structure-related variables not in isolation but in conjunction, while preserving the relationships among them.

Field observations, trap monitoring, and expert assessments provide important information for monitoring insect pests. However, since these methods require regular monitoring across large production areas, they can pose significant challenges in terms of time and labor [7]. In order to reduce this limitation, deep learning-based monitoring systems that automatically detect pests from trap images have been developed in recent years [16]. Although these systems, integrated with light and sticky traps, have been shown to provide high accuracy in garden conditions [17,18], these approaches focus on counting the pest from the image; they do not include the evaluation of environmental and structural variables that determine the density. Furthermore, evaluating the relationships between environmental conditions, tree characteristics, and temporal changes using traditional methods is not always easy [16]. This situation can make it difficult to detect changes in pest density promptly and to accurately identify high-risk periods. Therefore, supplementing field observations with prediction methods capable of analyzing multidimensional data is crucial for determining pest density more reliably and promptly [7,14].

In line with this need, machine learning methods offer an effective approach for identifying the complex relationships among the numerous variables that influence pest density [8,19]. These methods can predict the presence or density levels of pests by leveraging patterns learned from past observations. Indeed, previous studies that evaluated temperature, relative humidity, plant phenology, and other environmental variables together have demonstrated that the emergence times and population levels of insect pests can be predicted using machine learning methods [7,14,15]. These studies have reported that using lagged meteorological variables improves forecasting accuracy; for example, 20–35 day lagged temperature and humidity data in orchards show a high correlation with population density [20,21]. XGBoost, one of the methods that can be used for this purpose, stands out in the analysis of multidimensional agricultural data because it can model nonlinear relationships and feature interactions among variables [22]. However, obtaining reliable results from a powerful classification model such as XGBoost depends on properly addressing the class distribution in the data and the hyperparameters that determine the model’s learning process.

Random cross-validation can yield overly optimistic performance estimates in datasets where observations are not independent of each other, particularly when repeated observations from the same unit are distributed across training and validation folds [23,24]. Additionally, XGBoost prediction performance depends on selecting appropriate values for the hyperparameters that determine the model structure and learning process. Particle Swarm Optimization, used to find effective solutions in large and complex hyperparameter spaces, guides the search process by leveraging the individual and collective experiences of candidate solutions [25,26,27]. The mutation mechanism added to this method aims to reduce the likelihood of premature convergence by preserving particle diversity [28].

PSO variants enhanced with mutations and similar diversity conservation mechanisms have been shown in various application areas to yield better results in determining XGBoost hyperparameters compared to grid and random search [26,27,29]. However, some comparisons conducted on agricultural data have also reported that Bayesian optimization approaches provide higher performance than PSO and genetic algorithms, indicating that the choice of optimization method depends on the problem [30]. While high classification performance is important in pest density prediction, it is also necessary to understand which variables and thresholds the model uses to make decisions. Particularly in agricultural decision-support applications, the ability of domain experts to interpret predictions is crucial for the reliability and practical applicability of model results [31,32]. Therefore, translating complex model decisions into understandable IF–THEN rules can help ensure both prediction performance and interpretability [33]. In methods for deriving rules from tree communities, it is emphasized that the obtained rule set should be evaluated not only in terms of its accuracy but also in terms of criteria such as its coverage/support, fidelity to the original model, and the number and length of rules [34,35,36]. The studies summarized above demonstrate that pest populations can be estimated using environmental data. However, the reviewed literature indicates that most previous studies focus on individual methodological components, while limited attention has been given to integrating population-based hyperparameter optimization, robust model evaluation, and the extraction of interpretable rules from model decisions within a unified framework. To our knowledge, an interpretable pest density classification that considers environmental, temporal, and tree structure variables together in almond orchards has not been addressed previously. The contribution of this study is to combine these components within a unified validation framework designed to minimize information leakage during hyperparameter optimization and performance evaluation, while transforming the resulting model decisions into rules that can be examined by field experts. In this context, the contribution is at the level of application and integration, not methodology.

Accordingly, this study developed an explainable hybrid machine learning approach to predict pest insect density in almond orchards based on environmental, temporal, and tree structure-related variables. The proposed approach integrates Mutated Particle Swarm Optimization to determine XGBoost hyperparameters and rule extraction to make model decisions understandable within a unified framework. The key contributions of this study to the literature and practice are summarized below:

- Hyperparameter optimization with MPSO: The key hyperparameters of the XGBoost model were determined using Mutated Particle Swarm Optimization (MPSO), which aims to preserve particle diversity during the search process and reduce the likelihood of premature convergence. This enabled an effective search across the extensive hyperparameter space.
- Integrated evaluation of multidimensional data: In estimating pest density, environmental and meteorological variables were evaluated alongside temporal characteristics and structural features such as tree age, height, and canopy width. This ensured a comprehensive modeling of the relationships among the various variables affecting pest density.
- Combining prediction performance and interpretability: The proposed approach was compared with 16 different classification algorithms under the same evaluation conditions and achieved an average accuracy of 79.63% and an average Weighted F1-Score of 79.79%. Furthermore, by deriving IF–THEN rules from the XGBoost model’s decision structure—which include thresholds related to environmental, temporal, and tree structure factors—the model’s predictions were made examinable and interpretable by domain experts.

## 2. Related Studies

Studies aimed at predicting the emergence times and population levels of pest insects have found that variables related to meteorological conditions and plant growth are widely used. Skawsang et al. [15] estimated the population density of rice pests by analyzing meteorological observations and plant phenology data derived from satellite imagery using artificial neural networks, Random Forest, and multiple linear regression methods. Similarly, Marković et al. [7] modeled the daily emergence of insect pests using temperature and relative humidity data and various machine learning methods, demonstrating that the combined evaluation of environmental changes over several days improved prediction performance. Ibrahim et al. [14] on the other hand, used temperature, relative humidity, precipitation, and plant growth stages to estimate pest populations in orchards using a fuzzy neural network. These studies demonstrate that pest density can be estimated more reliably not only based on current insect counts but also by jointly evaluating environmental and phenological variables that influence population dynamics [7,15]. Similar approaches have also been applied in orchards. Pimentel et al. [20] determined that 35-day lagged meteorological data gave the highest prediction success in their artificial neural network model trained with eight years of observational data in apple orchards. Oliveira et al. [21] obtained a correlation of 0.865 with 20-day lagged meteorological variables on five years of pest density data in cotton. Rathod et al. [37] compared count-based time series models with machine learning methods and statistically confirmed the superiority between the models using the Diebold–Mariano test. Reddy et al. [38] and Minruhi et al. [39] showed that two-stage hybrid structures provided higher performance compared to single models.

When studies specific to almond orchards are examined, it is seen that the literature mainly focuses on pest biology, population monitoring and control strategies. Daane et al. [4] revealed the seasonal population dynamics of *Leptoglossus zonatus* in almond and pistachio orchards with a one-year monitoring and stated that calendar-based spraying programs were insufficient for this pest. Mamay et al. [5]. In their study conducted with sexual attractant traps in almond orchards in Türkiye, they determined the population peaks and activity period of the pest and emphasized that the control should be planned according to these periods. Broadhead et al. [40] evaluated the contribution of seasonal pest abundance measurements in predicting harvest period damage in commercial almond orchards; It was reported that although there was a strong relationship between abundance and damage, abundance data obtained with current sampling protocols did not increase the prediction accuracy, and almond variety characteristics were stronger determinants. This finding indicates that pest counts alone are not sufficient for reliable prediction in almond orchards; It shows that environmental and structural variables should also be included in the model. As a matter of fact, Callahan et al. [2] and Achhami et al. [12] revealed that species composition and sampling method in almond orchards differ significantly from garden to garden. Golan et al. [41] states that monitoring changes in pest density and reporting them to the producer in a timely manner is a basic requirement in integrated control. When examining the methods used to estimate pest populations, it is evident that machine learning methods capable of modeling nonlinear relationships and interactions among variables are gaining prominence. Singh et al. [42] estimated the yellow stem borer population in rice by combining artificial neural networks, support vector regression, k-nearest neighbors, and Random Forest models within a dynamic community structure. The study utilized minimum and maximum temperatures, precipitation, and morning and evening relative humidity values; it was determined that the proposed dynamic ensemble model provided higher prediction performance compared to individual models and both fixed-weight and unweighted ensemble methods [42]. This finding demonstrates that combining the strengths of different machine learning models can contribute to modeling complex changes in pest populations. One of these methods, XGBoost, is capable of modeling nonlinear relationships and complex interactions among variables thanks to its regularized tree-boosting architecture, and it produces particularly strong results on tabular data [22,43]. In studies combining PSO with XGBoost for agricultural regression problems, it has been shown that determining hyperparameters through heuristic optimization can support model performance [44]. In this context, it has been shown that the developed adaptive and mutation-based PSO variants reduce the risk of getting stuck in the local optimum while preserving particle diversity and provide competitive results compared to grid search, random search, and Bayesian methods in determining XGBoost hyperparameters [26,27]. Demir and Şahin [29] directly compared the gradient boost models run with default parameters with their versions optimized with PSO and separated the contribution of optimization. On the other hand, Dada et al. [30] reported that Bayesian optimization gave higher performance compared to PSO and genetic algorithm in an agricultural prediction problem; this shows that the contribution of the optimization method depends on the problem and data structure. However, in addition to prediction performance, class distribution, the reliability of the validation process, and the interpretability of model decisions are also important considerations for application-oriented models.

In addition to reliably evaluating model performance, the ability to explain how the obtained predictions are generated is important for agricultural decision support systems. Although ensemble models such as XGBoost can provide high prediction performance, the results they produce may be difficult to interpret directly due to their structure consisting of a large number of decision trees. Explainable Artificial Intelligence methods aim to reduce this limitation by identifying the variables that influence model predictions and their roles in the decision-making process [31,32]. Ensuring explainability in agricultural applications contributes to the evaluation of model results based on domain knowledge and facilitates their use by decision-makers [32]. In this context, converting decisions derived from tree-based models into IF–THEN rules is considered a suitable approach that allows complex prediction processes to be expressed through clear conditions and thresholds [33].

In agricultural practices, explainability is said to enable comparison of model results with field data and increase confidence among decision-makers [45,46]. Methods for rule extraction from tree populations involve combining, simplifying, and pruning rules; the performance of the resulting rule set is evaluated not only by accuracy but also by criteria such as coverage/support, fidelity to the original model, and the number and length of rules [34,35,36]. Reporting these criteria together prevents misleading interpretations of rules that have high accuracy but cover very few observations [34].

Prior to machine learning methods, the commonly used approach for predicting pest populations was degree-day and phenology models based on temperature accumulation. These models predict adult emergence and flight peak periods based on pest development threshold and biofixation history, and are used in planning the timing of control measures [47,48]. Rincon et al. [49] showed that combining phenology models with trap count data improved intraseason population prediction. While these approaches are strong in timing prediction, they do not directly include variables affecting pest density, such as tree structure and habitat, and are mostly parameterized for a single species. Machine learning-based classification approaches, on the other hand, offer a complementary option to these methods in terms of being able to evaluate numerous variables together and model nonlinear interactions between variables. A general review of the relevant studies reveals that meteorological and phenological variables are used to estimate pest populations, while machine learning methods have proven effective in modeling the complex relationships among these variables [7,14,15]. However, most existing studies focus on prediction performance and do not address hyperparameter optimization, rigorous separation of model tuning from evaluation, and the interpretability of model decisions within an integrated framework. Table 1 summarizes a comparison of the studies examined in terms of approach, variable groups used, problem type, validation design, and explainability dimensions. In particular, there are limited studies on interpretable pest density classification that jointly evaluate environmental, temporal, and tree-structure-related variables in almond orchards. In this study, an integrated approach combining hyperparameter optimization via MPSO, XGBoost classification, and rule extraction in an IF–THEN format is proposed. The aim is not only to predict pest density but also to ensure that model decisions can be examined and interpreted by domain experts.

## 3. Materials and Methods

The flowchart presented in Figure 1 illustrates the proposed explainable hybrid machine learning approach, which integrates nested stratified 5-fold cross-validation, Mutated Particle Swarm Optimization (MPSO)-based XGBoost hyperparameter optimization, performance evaluation on unseen outer validation folds, and explainable rule extraction. Hereafter, the term “proposed approach” refers to the overall framework, whereas the term “model” specifically refers to the trained XGBoost classifier. In the first stage, the dataset is loaded and preprocessed, including missing-value handling, numerical conversion of temporal variables, and encoding of the categorical target variable. The dataset is then divided using an outer stratified 5-fold cross-validation scheme, in which four folds are used for training and the remaining fold is retained as unseen validation data in each iteration. Within each outer training set, an inner stratified 5-fold cross-validation procedure is employed for MPSO-based hyperparameter optimization. A population of 100 particles explores the XGBoost hyperparameter space, including the number of estimators, maximum tree depth, learning rate, subsampling ratio, feature subsampling ratio, and gamma. For each candidate parameter set, the mean weighted F1-Score across the five inner validation folds is used as the fitness value. During the optimization process, the personal best (pBest) and global best (gBest) positions are updated, followed by adaptive inertia adjustment, velocity and position updates, and mutation. The optimization continues until the maximum number of iterations is reached or the early-stopping criterion is satisfied. The best hyperparameters obtained for each outer fold are then used to train an XGBoost classifier on the corresponding outer training set, and its performance is evaluated on the unseen outer validation fold. Finally, the results from all five outer folds are aggregated to calculate the mean and standard deviation of the performance metrics, and explainable IF–THEN decision rules are derived from the trained XGBoost decision structure to provide transparent and interpretable predictions (Figure 1).

### 3.1. Dataset Description

- The dataset used in this study contains a total of 1645 observations and 16 variables, comprising 15 predictor features and one target class variable. The predictor variables represent environmental and meteorological conditions, tree morphological characteristics, and temporal information. The target variable, Class, has a three-class structure consisting of Low (L), Medium (M), and High (H) pest density levels. The predictor variables are grouped into three main categories:
- Environmental and Meteorological Attributes: Altitude, Temperature, Humidity, Wind Speed, and Habitat.
- Tree Morphological Features: TreeAge, TreeHeight, and TreeCanopyWidth.
- Temporal Attributes: Year (2020–2022), Month, Day, Day_of_Year, Week, Day_of_Week, and Quarter.

Detailed definitions of all attributes in the dataset, together with their data types and descriptive statistical summaries (minimum, maximum, mean, and median values), are presented in Table 2.

The field data were collected between 2020 and 2022 from almond orchards located in Keban District and Nimri Village of Elazığ Province and Eğil District of Diyarbakır Province, Türkiye. Field observations were conducted through direct in situ visits to the selected orchards. Depending on pest activity and field conditions, the orchards were monitored through daily, weekly, and monthly inspections during the study period. Pest density was determined based on the abundance of *Cimbex quadrimaculata* larvae observed on different almond trees. Overall, the dataset comprises observations collected across three study locations during the 2020–2022 sampling period.

For the classification task, the target variable was defined according to the observed number of *C. quadrimaculata* larvae. Observations with 0–2 larvae were assigned to the Low (L) class, those with 3–5 larvae to the Medium (M) class, and those with 6 or more larvae to the High (H) class. These categories represent relative pest density levels within the present dataset and should not be interpreted as universally applicable economic injury thresholds. The resulting class distribution is presented in Table 3.

As shown in Table 3, Class L accounts for 39.51% of the observations, while Classes H and M represent 31.31% and 29.18%, respectively. The three classes are therefore represented at moderately different frequencies in the dataset.

### 3.2. Data Preprocessing and Nested Stratified 5-Fold Cross-Validation

To improve model generalizability and provide an unbiased estimate of predictive performance, the raw dataset was first cleaned, temporal features were converted to numerical formats, and the categorical target variable was numerically encoded. Model development and evaluation were then conducted using nested stratified 5-fold cross-validation. The outer 5-fold cross-validation was used for performance evaluation, while the inner 5-fold cross-validation was performed exclusively within each outer training fold for MPSO-based hyperparameter optimization. Stratification was applied to maintain approximately the original class distribution across the folds.

### 3.3. Implementation Details and Reproducibility

The analytical pipeline was implemented as a nested stratified cross-validation framework to ensure a clear separation between hyperparameter optimization and performance evaluation. First, the dataset was loaded and preprocessed, and the categorical target variable was numerically encoded using a LabelEncoder. The predictor matrix (X) and target vector (y) were then constructed. Model development and evaluation were performed using an outer Stratified 5-Fold Cross-Validation (CV) procedure with shuffling enabled and a random state of 42. In each outer iteration, four folds were used as the outer training set, while the remaining fold was retained as unseen validation data and was not involved in hyperparameter optimization.

Hyperparameter optimization was performed exclusively on the outer training set using Mutated Particle Swarm Optimization (MPSO). Within each outer training fold, an inner Stratified 5-Fold CV procedure was employed to evaluate candidate XGBoost hyperparameter configurations. The optimization search space comprised six XGBoost hyperparameters: n_estimators (50–300), max_depth (2–8), learning_rate (0.01–0.30), subsample (0.50–1.00), colsample_bytree (0.50–1.00), and gamma (0.00–5.00). The n_estimators and max_depth parameters were rounded to integer values when converting particle positions into XGBoost parameter configurations. For each candidate configuration, the mean Weighted F1-score obtained across the five inner validation folds was used as the fitness function. Thus, the outer validation observations were completely excluded from the MPSO and hyperparameter-selection process.

The MPSO procedure used a population of 100 particles and a maximum of 50 iterations. The inertia weight was adaptively decreased from 0.90 to 0.40 during optimization, while both the cognitive (c1) and social (c2) acceleration coefficients were set to 2.0. Mutation was applied with a probability of 0.20 and a mutation strength of 0.10 to increase population diversity and reduce premature convergence. The personal best (pBest) and global best (gBest) solutions were updated according to the fitness values obtained during each iteration. An early-stopping mechanism with a patience of five iterations and a minimum improvement threshold (min_delta) of 1 × 10^−4^ was used to terminate optimization when no meaningful improvement in the global best fitness was observed.

After MPSO was completed for an outer fold, the hyperparameter configuration associated with the best inner-CV fitness was selected. An XGBoost classifier was then trained using the complete outer training set with these optimized parameters and subsequently evaluated on the corresponding unseen outer validation fold. This procedure was independently repeated for all five outer folds. Model performance was assessed using accuracy, weighted precision, weighted recall, Weighted F1-Score, and log loss. Predictions from the outer validation folds were also consolidated to construct an out-of-fold confusion matrix and classification report. The mean and standard deviation of the performance metrics across the five outer folds were reported to characterize both predictive performance and its variability.

The complete sequence of data preprocessing, outer-fold separation, inner-fold hyperparameter optimization, XGBoost training, unseen outer-fold evaluation, aggregation of performance metrics, and subsequent explainable rule extraction is summarized in Figure 1. This nested design ensures that hyperparameter selection is performed independently of the observations used for final performance estimation, thereby reducing optimistic bias and providing a more rigorous assessment of model generalization.

Computational Environment: All analyses were implemented in Python 3.11.11 using the Spyder integrated development environment (IDE) on a 64-bit Windows 11 Pro operating system. The experiments were conducted on a computer equipped with an Intel Core i5-10400 CPU running at 2.90 GHz and 8 GB of RAM. The system included integrated Intel UHD Graphics 630; however, GPU acceleration and CUDA were not used, and all analyses were executed on the CPU. The principal Python packages used in the analysis were NumPy 1.26.4, pandas 3.0.1, scikit-learn 1.8.0, and XGBoost 3.1.2.

### 3.4. Extraction of Explainable Decision Rules

In line with the Explainable Artificial Intelligence (XAI) approach, an explainable rule-extraction procedure was applied to make the decision structure of the XGBoost classifier more transparent and interpretable. The decision rules were derived directly from the tree structures generated by the trained XGBoost model. Each decision tree consists of a sequence of internal decision nodes defined by feature-specific split thresholds and terminal leaf nodes representing the resulting model decisions. To obtain interpretable rules, the decision paths within the trees were examined from the root node to the corresponding terminal leaf nodes. The successive feature–threshold conditions encountered along each decision path were combined to construct the antecedent (IF) part of a rule, while the decision associated with the corresponding terminal path was represented as the consequent (THEN) part.

When a feature appeared more than once along the same decision path, its successive threshold conditions were combined to obtain a bounded feature interval where applicable. In this way, multiple tree-splitting conditions along a path could be represented in a compact interval-based form. The extracted rules therefore describe combinations of feature intervals associated with the model’s classification decisions and follow the general form:

(1) $$IF\left(L_{1}\leq X_{1}\leq U_{1}\right)AND\left(L_{2}\leq X_{2}\leq U_{2}\right)\ldots \left(L_{k}\leq X_{k}\leq U_{k}\right)THEN\;Class=C$$

where $X_{k}$ represents a predictor involved in the decision path; $L_{k}$ and $U_{k}$ denote its lower and upper threshold bounds, respectively; and C represents the corresponding predicted class. This procedure converts the tree-based decision structure learned by XGBoost into explicit symbolic IF–THEN rules, allowing the combinations of feature conditions underlying the model’s predictions to be examined in a more transparent and human-interpretable form.

Rule accuracy was calculated by applying each extracted rule to the dataset and identifying all observations that satisfied the rule conditions. For a given rule, accuracy was defined as the proportion of observations satisfying the rule conditions whose actual class label matched the class predicted by the rule. Specifically, if $N_{r}$ denotes the total number of observations satisfying a rule and $N_{c}$ denotes the number of those observations whose actual class corresponds to the rule-predicted class, rule accuracy was calculated as follows:

(2) $$Rule\;Accuracy=\frac{N_{c}}{N_{r}}$$

where $N_{r}$ is the total number of observations satisfying the rule conditions, and $N_{c}$ is the number of those observations whose actual class label matches the class predicted by the rule.

## 4. Results

The proposed MPSO-optimized XGBoost classification approach (the proposed method) was compared with 16 classification algorithms under a Stratified 5-Fold Cross-Validation protocol. The mean accuracy and mean Weighted F1-Score values, together with their standard deviations across the five folds, are presented in Figure 2.

As shown in Figure 2, the proposed method achieved a mean accuracy of 79.63% and a mean Weighted F1-Score of 79.79%. Standard XGBoost achieved corresponding values of 79.21% and 79.23%, respectively. Thus, the proposed method showed numerically higher mean values than standard XGBoost, with differences of 0.42 percentage points in accuracy and 0.56 percentage points in Weighted F1-Score. However, these differences were relatively small, and variability was observed across the cross-validation folds. Therefore, in the absence of a formal statistical significance test, the observed differences should be interpreted as modest numerical improvements rather than evidence of statistically significant superiority.

Other ensemble and machine learning methods also showed comparable classification performance, including CatBoost (78.91%), ExtraTrees (77.39%), Random Forest (77.20%), MLP (77.33%), and TabNet (76.72%). In comparison, the evaluated rule-based algorithms, including RIPPER, C4.5 Rules, IREP, and RuleFit, produced accuracy values ranging from 70.46% to 74.41%. Beyond predictive performance, the proposed approach incorporates an explainability component in which the decision structures learned by the XGBoost classifier are represented as interpretable IF–THEN rules. As described in the rule-extraction methodology, feature–threshold conditions along the decision paths of the XGBoost trees were converted into interval-based symbolic rules. This representation provides an interpretable view of the feature conditions underlying the model’s classification decisions while maintaining classification performance comparable to the best-performing methods evaluated in this study.

The performance of the proposed MPSO-optimized XGBoost approach was further evaluated using the consolidated confusion matrix (Figure 3a), class-specific performance metrics (Figure 3b), and performance variation across the five outer cross-validation folds (Figure 3c). The consolidated confusion matrix, generated by aggregating the out-of-fold predictions from the five outer validation folds, provides an overall representation of the classification results. The proposed approach achieved a mean accuracy of 79.27 ± 1.66% and a mean Weighted F1-Score of 79.29 ± 1.38% across the five outer folds.

The class-specific results presented in Figure 3b show that classification performance varied among the three classes. For the H (High) class, precision, recall, and F1-score were 84.78%, 85.44%, and 85.11%, respectively. The L (Low) class achieved a precision of 85.44%, recall of 83.08%, and F1-score of 84.24%. Comparatively lower performance was observed for the M (Medium) class, with a precision of 65.59%, recall of 67.50%, and F1-score of 66.53%, indicating that this intermediate category was more difficult to distinguish than the H and L classes.

Figure 3c further illustrates the variation in accuracy, Weighted F1-Score, and log loss across the five outer cross-validation folds. Accuracy and Weighted F1-Score followed relatively similar patterns across the folds, with mean values of 79.27 ± 1.66% and 79.29 ± 1.38%, respectively, while the mean log loss was 0.5360 ± 0.0913. These fold-level results indicate relatively limited variation in accuracy and Weighted F1-Score across the outer validation subsets, although greater variability was observed in log loss. Overall, the results demonstrate relatively consistent predictive performance across the five outer folds while also revealing clear class-specific differences, particularly the comparatively lower classification performance observed for the M class.

In line with the Explainable Artificial Intelligence (XAI) approach, the symbolic decision rules derived from the optimized XGBoost model, together with their accuracy values, are presented in Table 4. These rules provide an interpretable representation of the feature–threshold combinations underlying the model’s classification decisions. Examination of the extracted rules showed that combinations of temporal (Day_of_Year, Month, Day), meteorological (Temperature, Humidity, Wind Speed, Altitude), and morphological (TreeAge, TreeHeight, TreeCanopyWidth) features were associated with the classification of pest density levels through specific threshold ranges.

The decision rules representing Class L (Low Density) have high accuracy rates ranging from 0.96 to 0.99 (Table 4). These results indicate that the extracted rules identify relatively distinct feature–threshold combinations associated with Class L. In particular, during the later part of the season (185 ≤ Day_of_Year ≤ 206, i.e., mid- to late July) and at higher temperature values (28.5 °C ≤ Temperature ≤ 36.7 °C), observations satisfying specific tree height (2.0–4.5 m) or canopy width (1.4–2.8 m) conditions were classified as Class L with rule accuracies ranging from 0.96 to 0.99. These patterns indicate that combinations of seasonal timing, temperature, and morphological characteristics were associated with the model’s classification of observations as Class L. These relationships should be interpreted as model-derived predictive associations rather than biologically established thresholds or causal relationships.

The rules derived for Class H (High Density) exhibit accuracy rates ranging from 0.84 to 0.90 (Table 4). Tree age (8 ≤ TreeAge ≤ 20), seasonal timing (139 ≤ Day_of_Year ≤ 178, i.e., late May–late June), and temperature (22.2 °C ≤ Temperature ≤ 36.7 °C) frequently appeared in the extracted rules associated with Class H. The co-occurrence of these feature ranges within the rules indicates that combinations of tree age, seasonal timing, and temperature contributed to the model-derived predictive patterns associated with Class H. As with Class L, these thresholds represent the decision structure learned by the XGBoost model and should not be interpreted as causal, mechanistic, or universally applicable agronomic thresholds.

The accuracy rates of the rules defining Class M (Medium Density) were lower, ranging from 0.60 to 0.67 (Table 4). This finding is consistent with the comparatively lower class-specific performance observed in the confusion matrix (Figure 3a) and performance metrics (Figure 3b). The extracted rules for Class M involve combinations of morphological, temporal, and environmental variables whose ranges may overlap with patterns associated with Classes L and H. Therefore, these rules should be interpreted as model-derived predictive associations for the intermediate density category rather than as evidence of a biological or temporal transition stage or an operational early-warning threshold. Nested Cross-Validation Analysis

To further assess the generalization performance of the proposed MPSO-XGBoost approach while separating hyperparameter optimization from model evaluation, an additional nested stratified 5-fold cross-validation analysis was performed. In each outer fold, MPSO-based hyperparameter optimization was conducted exclusively within the corresponding outer training set using an inner stratified 5-fold cross-validation procedure, with Weighted F1-score used as the optimization fitness criterion. The optimized model was subsequently trained on the complete outer training set and evaluated on the unseen outer validation fold.

As shown in Table 5, across the five outer folds, the proposed approach achieved a mean accuracy of 0.7927 ± 0.0166 and a mean Weighted F1-Score of 0.7929 ± 0.0138. The mean precision and recall were 0.7967 ± 0.0147 and 0.7927 ± 0.0166, respectively, while the mean log loss was 0.5360 ± 0.0913. The relatively limited variation in accuracy and Weighted F1-Score across the outer folds indicates that the predictive performance was reasonably consistent across the five unseen validation subsets.

The MPSO search space was defined as follows: n_estimators = 50–300, max_depth = 2–8, learning_rate = 0.01–0.30, subsample = 0.50–1.00, colsample_bytree = 0.50–1.00, and gamma = 0.00–5.00. The MPSO algorithm was configured with a population of 100 particles and a maximum of 50 iterations. The inertia weight was adaptively decreased from 0.90 to 0.40 during optimization, while the cognitive and social acceleration coefficients were set to c1 = 2.0 and c2 = 2.0, respectively. To improve exploration of the search space, the mutation rate (probability) was set to 0.20, with a mutation strength of 0.10. Early stopping was applied when no improvement greater than 1 × 10^−4^ was observed for five consecutive iterations. The optimal XGBoost hyperparameters obtained independently for each outer fold are presented in Table 6.

The number of estimators selected across the five outer folds ranged from 139 to 300, while the maximum tree depth ranged from 3 to 8. The optimized learning rate ranged from 0.0123 to 0.2614, the subsample ratio from 0.5000 to 1.0000, the colsample_bytree ratio from 0.5000 to 0.9013, and gamma from 0.0000 to 0.4415. The corresponding inner-CV Weighted F1-scores ranged from 0.7795 to 0.7911. The variation in the selected hyperparameters across the outer folds reflects the independent optimization performed within each outer training set. The corresponding outer validation fold was not used during hyperparameter selection.

## 5. Discussion

In this study, an explainable hybrid machine learning framework combining Mutated Particle Swarm Optimization (MPSO)-based hyperparameter optimization, XGBoost classification, and explainable rule extraction was evaluated for the classification of pest density levels based on environmental, morphological, and temporal variables. In the stratified 5-fold cross-validation experiment used for comparison with the 16 benchmark classification algorithms, the proposed approach achieved a mean accuracy of 79.63% and a mean Weighted F1-Score of 79.79%. These results were compared with those obtained from 16 classification algorithms commonly used in machine learning studies.

Standard XGBoost achieved a mean accuracy of 79.21% and a mean Weighted F1-Score of 79.23%. Thus, the proposed MPSO-XGBoost approach produced numerically higher mean values, corresponding to differences of 0.42 percentage points in accuracy and 0.56 percentage points in Weighted F1-Score. However, these differences were relatively small, and variability was observed across the cross-validation folds. Because no formal statistical significance test was performed for these comparisons, the observed differences should be interpreted as modest numerical differences rather than evidence of statistically significant superiority. Other ensemble methods, including CatBoost (78.91% accuracy) and Random Forest (77.20% accuracy), also demonstrated comparable classification performance.

In addition to the benchmark comparison, nested stratified 5-fold cross-validation was used to provide a more rigorous assessment in which hyperparameter optimization was performed within the inner folds and predictive performance was evaluated on unseen outer validation folds. Across the five outer folds, MPSO-XGBoost achieved a mean accuracy of 79.27 ± 1.66% and a mean Weighted F1-Score of 79.29 ± 1.38%. The similarity between these nested cross-validation results and those obtained in the standard stratified 5-fold comparison suggests that the observed predictive performance was reasonably stable under the more stringent nested evaluation procedure.

### 5.1. MPSO-Based Hyperparameter Optimization of XGBoost

In the proposed framework, MPSO was used to optimize the XGBoost hyperparameters, including the number of estimators, maximum tree depth, learning rate, subsampling ratio, feature subsampling ratio, and gamma. The mutation mechanism incorporated into the particle swarm optimization process was used to increase exploration of the predefined hyperparameter search space. The mean Weighted F1-Score across the inner validation folds was used as the fitness criterion during MPSO. The resulting optimized parameter configurations were subsequently used to train the XGBoost classifier.

The relatively small numerical differences between MPSO-XGBoost and standard XGBoost indicate that the contribution of MPSO should be interpreted cautiously. Under the applied evaluation protocol, MPSO-XGBoost achieved slightly higher mean accuracy and Weighted F1-Score values than standard XGBoost. However, because no formal statistical significance test was performed, these differences cannot be interpreted as evidence that MPSO consistently improves predictive performance.

### 5.2. Class-Based Performance and Feature Complexity Analysis

The class-specific metrics presented in Figure 3b indicate differences in classification performance among the three pest density categories. Based on the consolidated out-of-fold predictions from the nested cross-validation analysis, the H (High) class achieved an F1-score of 85.11%, while the L (Low) class achieved an F1-score of 84.24%. Comparatively lower performance was observed for the M (Medium) class, with a precision of 65.59%, a recall of 67.50%, and an F1-score of 66.53%. These results indicate that the intermediate M category was more difficult for the model to distinguish from the other two classes.

The confusion matrix (Figure 3a) further shows that a considerable proportion of the classification errors involved the M class, including 86 M observations classified as L and 70 M observations classified as H. Although the extracted rules for the M category include feature ranges that may overlap with those observed for the L and H categories, these patterns should be interpreted as model-derived predictive associations rather than evidence of a biological or temporal transition between pest density classes. Therefore, Class M is considered an intermediate classification category rather than a transitional stage. Future studies could investigate whether ordinal classification or fuzzy logic approaches provide a more suitable representation of the boundaries among these pest density categories.

The comparatively lower rule accuracy observed for the M class should also be considered when interpreting the explainable rule set. The extracted Class M rules achieved rule accuracy values ranging from approximately 0.60 to 0.67, indicating lower predictive accuracy for the extracted M-class rules than for the rules associated with the H and L categories. This finding represents a limitation of the current rule-based interpretation and suggests that the M category may contain less distinct or more overlapping predictive patterns. Further studies using additional data and validation across different seasons and locations are required to determine whether these patterns are reproducible.

### 5.3. Comparison with Previous Studies

Comparison with previous studies should be interpreted cautiously because the available studies differ in crop species, target pests, datasets, predictor variables, and prediction tasks. Marković et al. [7] demonstrated that temperature and relative humidity can support machine learning-based prediction of pest occurrence, while Ibrahim et al. [14] showed that combining meteorological variables with plant growth stages provides useful information for predicting pest population dynamics. Similarly, Pimentel et al. [20] reported that lagged meteorological variables contributed to the prediction of fruit fly populations in apple orchards, emphasizing the potential relevance of temporal information in addition to current environmental conditions.

In the present study, temperature and intra-seasonal temporal variables appeared in the extracted decision rules, indicating predictive associations between these variables and pest density categories. These findings are broadly consistent with previous studies emphasizing the relevance of meteorological and temporal information in pest prediction, although they should not be interpreted as evidence of causal relationships.

From a modeling perspective, Singh et al. [42] showed that combining multiple machine learning components within a dynamic ensemble improved pest population prediction compared with individual models, illustrating the potential value of integrated learning strategies for complex pest–environment relationships. In the context of almond orchards, Broadhead et al. [40] reported that pest abundance measurements alone did not improve end-of-season damage prediction and that cultivar- and orchard-related characteristics were stronger predictors. In the present study, tree age and tree height also appeared in the extracted decision rules, indicating that structural tree characteristics contributed to the model’s classification decisions.

These comparisons suggest that jointly considering meteorological, temporal, and crop structural information may provide useful predictive information for pest density classification. Nevertheless, the relative importance and generalizability of these associations require further evaluation across independent datasets, different growing seasons, and different geographical regions. The present study contributes to this research direction by integrating heterogeneous predictors with MPSO-optimized XGBoost classification and explainable IF–THEN rule extraction within a unified framework.

### 5.4. Balance Between Explainability and Predictive Performance

Traditional rule-based methods such as RIPPER, C4.5 Rules, IREP, and RuleFit provide transparent IF–THEN decision structures by design and achieved accuracy values ranging from 70.46% to 74.41% in the present experiments. Other machine learning approaches, including TabNet (76.72%) and MLP (77.33%), achieved different levels of predictive performance but generally provide less directly interpretable decision structures than explicit rule-based models.

The proposed framework addresses this interpretability challenge by deriving explicit IF–THEN rules from the decision structures learned by the XGBoost classifier. The extracted rules provide feature–threshold combinations derived from the XGBoost decision structures that can be examined by researchers and domain experts. For example, some extracted rules associated combinations of tree age and intra-seasonal temporal conditions with the H (High) category, whereas other rules associated combinations of temporal and temperature conditions with the L (Low) category. These relationships should be interpreted as model-derived predictive associations rather than causal or mechanistic relationships.

## 6. Conclusions

This study presented an explainable hybrid machine learning framework integrating Mutated Particle Swarm Optimization (MPSO)-based hyperparameter optimization, XGBoost classification, and explainable IF–THEN rule extraction for the classification of pest density levels in almond orchards. The framework jointly considered environmental, meteorological, temporal, and tree morphological variables to characterize Low (L), Medium (M), and High (H) pest density categories.

Under the applied evaluation protocol, the proposed MPSO-XGBoost approach achieved a mean accuracy of 79.63% and a mean Weighted F1-Score of 79.79%. Standard XGBoost achieved corresponding values of 79.21% and 79.23%, indicating modest numerical differences of 0.42 and 0.56 percentage points, respectively. Because no formal statistical significance test was performed, these differences should not be interpreted as evidence of statistically significant superiority. Instead, the results indicate that MPSO-XGBoost provided classification performance comparable to the strongest machine learning approaches evaluated in this study while enabling an additional rule-based interpretation of the model’s decision structure.

The explainability component converted feature–threshold combinations learned by XGBoost into explicit IF–THEN rules, providing an interpretable representation of the predictive associations underlying the classifications. The H and L categories showed stronger class-specific classification performance, whereas the intermediate M category was more difficult to distinguish. In particular, the lower rule accuracy observed for Class M (0.60–0.67) highlights a limitation of the current rule-based interpretation and indicates that the predictive patterns associated with this category were less distinct than those associated with the H and L categories. The extracted relationships should therefore be interpreted as model-derived predictive associations rather than causal or mechanistic relationships.

Overall, the findings demonstrate the potential of combining hyperparameter-optimized tree-based classification with interpretable rule extraction for pest density assessment in almond orchards. Nevertheless, the generalizability of the framework should be evaluated using independent datasets collected across additional growing seasons, geographical regions, and orchard conditions. Future studies could also investigate alternative approaches, including ordinal classification and fuzzy logic, to better characterize the intermediate M category and further improve the robustness and interpretability of pest density classification.

## Figures and Tables

**Figure 1:**
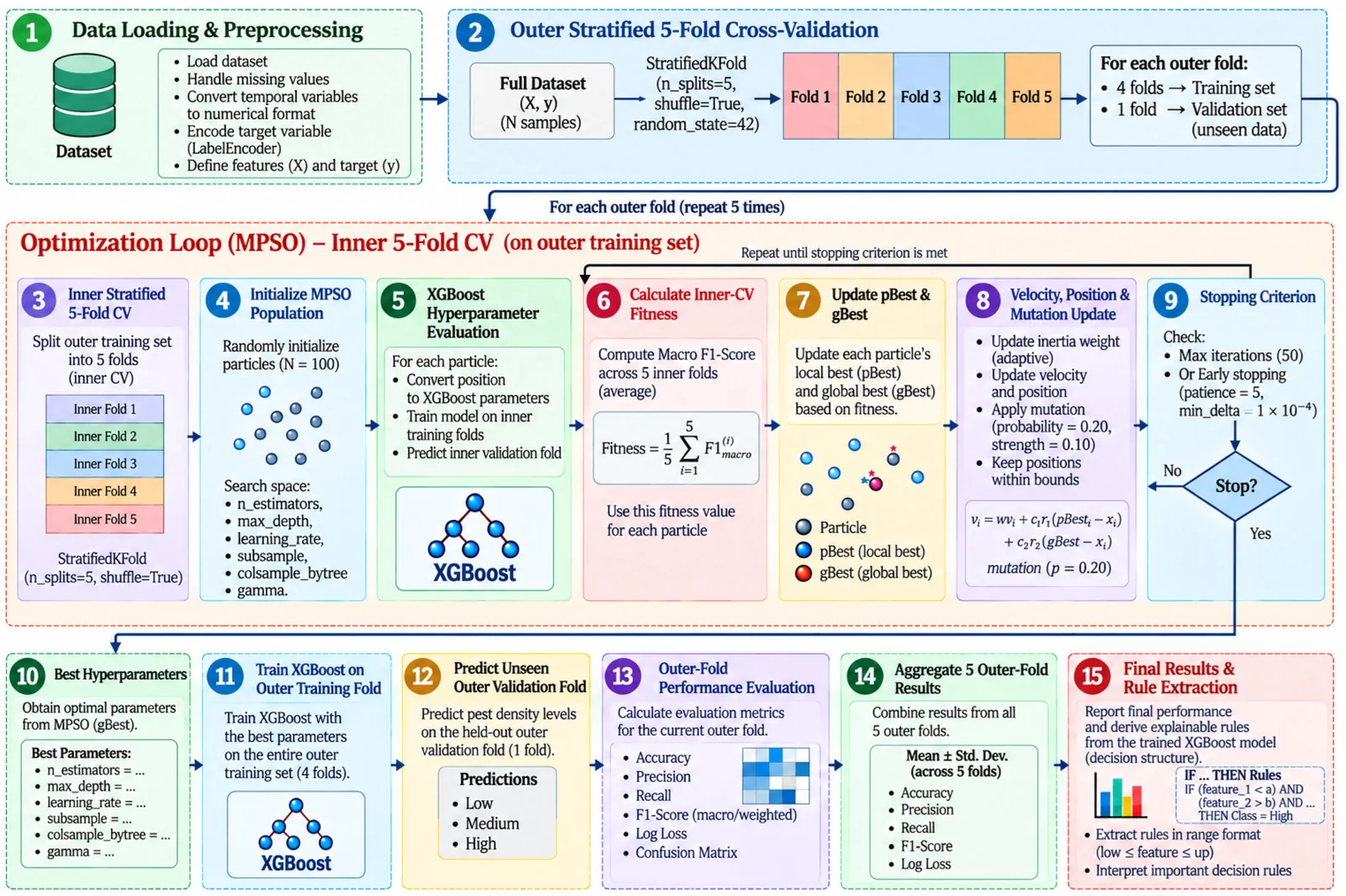
General workflow of the proposed explainable hybrid machine learning approach, including nested stratified 5-fold cross-validation (CV), Mutated Particle Swarm Optimization (MPSO)-based Extreme Gradient Boosting (XGBoost) hyperparameter optimization, outer-fold performance evaluation, and explainable rule extraction.

**Figure 2:**
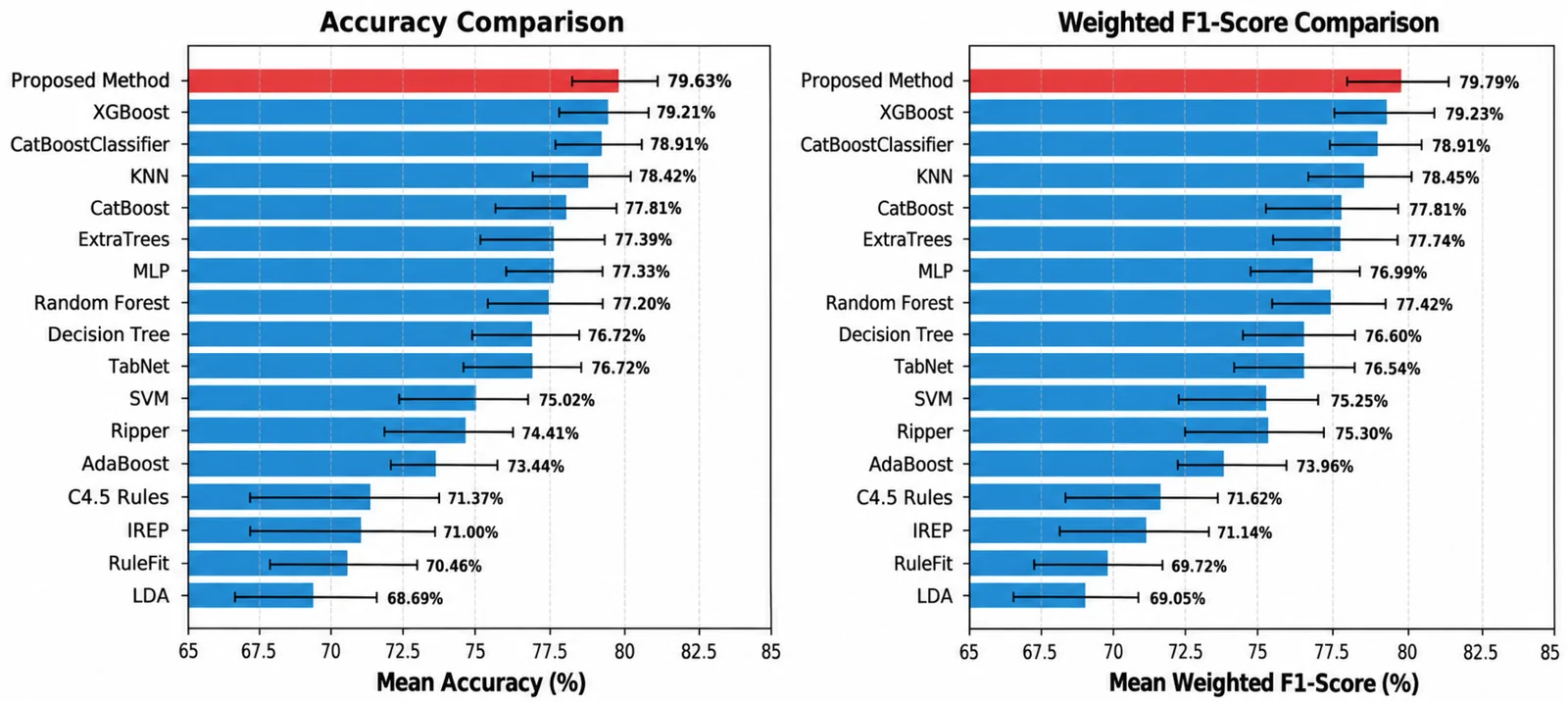
Comparison of the mean accuracy (%) and mean Weighted F1-Score (%) of the proposed MPSO-XGBoost approach and other classification models under stratified 5-fold cross-validation. Error bars represent ±1 standard deviation (SD) across the five cross-validation folds.

**Figure 3:**
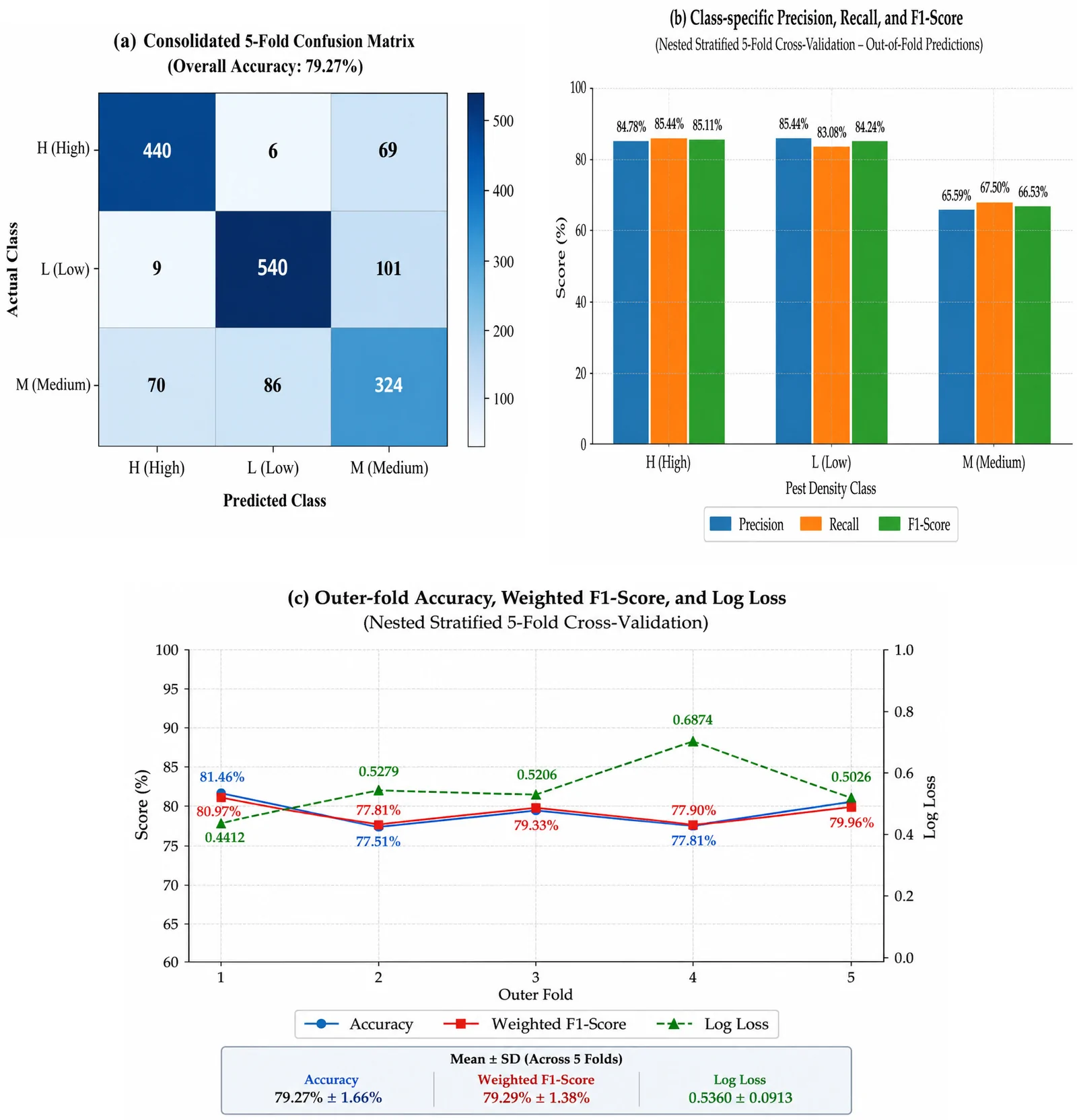
Detailed performance analysis of the proposed method: (**a**) Consolidated 5-Fold Cross-Validation Confusion Matrix, (**b**) Class-based performance metrics (Precision, Recall, F1-Score), and (**c**) the variation in cross-foldperformance metrics (accuracy, Weighted F1-Score, log loss).

**Table 1:** Comparison of selected studies on pest population/density prediction.

Study	Crop/Target Pest	Feature Groups	Method	Problem Type	Interpretability
[15]	Rice/brown planthopper	Ground meteorological observations, satellite-derived crop phenology	ANN, Random Forest, multiple linear regression	Regression	None
[7]	Field and orchard/various pest species	Temperature, relative humidity (multi-day)	Various machine learning methods	Classification (occurrence)	None
[14]	Orchard/pest population	Temperature, relative humidity, rainfall, plant growth stages	Fuzzy neural network	Regression	Partial (fuzzy rules)
[42]	Rice/yellow stem borer	Min–max temperature, rainfall, morning–evening relative humidity	Dynamic ensemble (ANN, SVR, kNN, RF)	Regression	None
[37]	Rice/Asian rice gall midge	Air temperature, relative humidity, rainfall, insolation	INGARCHX, ANNX, SVRX	Count time series	None
[38]	Rice/yellow stem borer	Temperature, relative humidity, sunshine hours, rainfall	Zero-inflated hybrid count models	Count time series	None
[39]	Groundnut/root grub, leaf miner, earwig	Exogenous weather variables	INGARCHX hybridised with ANN, SVR, ELM, RF	Count time series	None
[20]	Apple/*Anastrepha fraterculus*	Meteorological data with 35-day lag	ANN	Regression	None
[21]	Cotton/*Anthonomus grandis grandis*	Meteorological data with 20-day lag	ANN	Regression	None
[8]	Open field and high tunnel/*Frankliniella occidentalis*	16 environmental predictors	Random Forest, GBM, XGBoost	Prediction	None (variable importance only)
[19]	Longan/*Tessaratoma papillosa*	IoT-acquired meteorological data	LSTM combined with kNN, SVM, RF	Classification (damage severity)	None
[44]	Greenhouse/not a pest study	Meteorological variables	PSO-XGBoost	Regression	None
[40]	Almond/*Amyelois transitella*	Trap counts, cultivar characteristics, orchard management	Damage prediction model	Regression	None
[5]	Almond (Türkiye)/ *Trichoplusia ni*	Trap counts, seasonal calendar	Descriptive monitoring (no model)	-	-
This study	Almond/*Cimbex quadrimaculata*	Environmental and meteorological, tree morphological, temporal	MPSO-XGBoost + rule extraction	Classification (three classes)	IF–THEN rule

**Table 2:** Definitions and statistical summary of the features in the dataset.

Feature	Description/Unit	Min	Max	Average	Median
Altitude	Elevation (m)	842.00	1395.00	1124.55	1381.00
Temperature	Temperature (°C)	13.60	36.70	25.33	25.10
Humidity	Relative Humidity (%)	16.30	75.30	33.24	29.10
Wind Speed	Wind Speed (m/s)	1.00	5.00	2.04	2.00
TreeAge	Tree Age (Years)	4.00	20.00	9.67	8.00
Habitat	Habitat Type/Code	1.00	6.00	1.55	2.00
TreeHeight	Tree Height (m)	1.90	4.50	3.12	2.70
Tree Canopy Width	Canopy Width (m)	1.20	2.80	1.99	1.90
Year	Data Collection Year	2020	2022	2021	2021
Month	Month (4–7)	4.00	7.00	5.67	6.00
Day	Day of the Month (1–30)	1.00	30.00	15.76	17.00
Day_of_Year	Day of the Year	111.00	206.00	156.89	157.00
Week	Week of the Year	16.00	30.00	22.73	23.00
Day_of_Week	Day of the Week (0–6)	0.00	6.00	2.76	3.00
Quarter	Quarter of the Year	2.00	3.00	2.21	2.00

**Table 3:** Target Class Distribution.

Class	Number of Samples	Percentage (%)
L (Low)	650	39.51%
H (High)	515	31.31%
M (Medium)	480	29.18%
Total	1645	100.00%

**Table 4:** Explainable Decision Rules and Accuracy Rates Derived by the Proposed Method.

Rule	Class	Accuracy
IF 185 ≤ Day_of_Year ≤ 206 AND 28.5 ≤ Temperature ≤ 36.7 AND 7 ≤ Day ≤ 30 AND 2 ≤ TreeHeight ≤ 4.5	L	0.99
IF 7 ≤ Month ≤ 7 AND 7 ≤ Day ≤ 30 AND 1.4 ≤ TreeCanopyWidth ≤ 2.8	L	0.97
IF 7 ≤ Month ≤ 7 AND 7 ≤ Day ≤ 30 AND 2.1 ≤ TreeHeight ≤ 4.5	L	0.96
IF 143 ≤ Day_of_Year ≤ 178 AND 22.2 ≤ Temperature ≤ 36.7 AND 8 ≤ TreeAge ≤ 20	H	0.90
IF 143 ≤ Day_of_Year ≤ 174 AND 8 ≤ TreeAge ≤ 20	H	0.89
IF 4 ≤ Month ≤ 7 AND 139 ≤ Day_of_Year ≤ 174 AND 8 ≤ TreeAge ≤ 20	H	0.84
IF 1381 ≤ Altitude ≤ 1385 AND 27.9 ≤ Humidity ≤ 75.3 AND 0 ≤ Day_of_Week ≤ 5	M	0.60
IF 3 ≤ Wind Speed ≤ 5 AND 13.6 ≤ Temperature ≤ 29.3 AND 126 ≤ Day_of_Year ≤ 206 AND 4 ≤ TreeAge ≤ 8	M	0.67
IF 1.9 ≤ TreeHeight ≤ 2.3 AND 126 ≤ Day_of_Year ≤ 206 AND 18.2 ≤ Temperature ≤ 24.3	M	0.67

**Table 5:** Performance of MPSO-XGBoost across the outer folds of nested stratified 5-fold cross-validation.

Outer Fold	Accuracy	Precision	Recall	Weighted F1	Macro F1	Log Loss
1	0.8146	0.8100	0.8146	0.8097	0.8019	0.4412
2	0.7751	0.7835	0.7751	0.7781	0.7717	0.5279
3	0.7933	0.8119	0.7933	0.7982	0.7927	0.5206
4	0.7781	0.7801	0.7781	0.7790	0.7699	0.6874
5	0.8024	0.7981	0.8024	0.7996	0.7924	0.5026
Mean ± SD	0.7927 ± 0.0166	0.7967 ± 0.0147	0.7927 ± 0.0166	0.7929 ± 0.0138	0.7857 ± 0.0142	0.5360 ± 0.0913

**Table 6:** Optimal XGBoost hyperparameters identified by MPSO within each outer training fold.

Fold	n_estimators	max_depth	learning_rate	Subsample	colsample_bytree	Gamma	Inner-CV Weighted F1
1	295	3	0.213600	0.575551	0.500000	0.000000	0.7795
2	300	8	0.217715	1.000000	0.500000	0.312912	0.7903
3	207	8	0.012347	0.783515	0.901306	0.441461	0.7827
4	139	6	0.261444	0.804426	0.767818	0.000000	0.7848
5	144	3	0.230337	0.500000	0.712112	0.000000	0.7911

## Data Availability

The data presented in this study are available in the Supplementary Materials (Dataset S1) accompanying this article.

## Supplementary Materials

The following supporting information can be downloaded at https://www.mdpi.com/article/10.3390/insects17100996/s1, Dataset S1: Dataset used for pest density classification in almond orchards.

## Abbreviations

The following abbreviations are used in this manuscript:

MPSO	Mutated Particle Swarm Optimization
XAI	Explainable Artificial Intelligence
IPM	Integrated Pest Management
